# Deep learning–based scan range optimization can reduce radiation exposure in coronary CT angiography

**DOI:** 10.1007/s00330-023-09971-9

**Published:** 2023-08-08

**Authors:** Aydin Demircioğlu, Denise Bos, Ender Demircioğlu, Sahar Qaadan, Tobias Glasmachers, Oliver Bruder, Lale Umutlu, Kai Nassenstein

**Affiliations:** 1 Institute of Diagnostic and Interventional Radiology and Neuroradiology, University Hospital Essen, Hufelandstr. 55, 45147 Essen, Germany; 2 Department of Thoracic and Cardiovascular Surgery, West German Heart and Vascular Centre Essen, University Hospital Essen, 45147 Essen, Germany; 3 Department of Mechatronics and Artificial Intelligence Engineering, German Jordanian University, Madaba, JO-11180 Jordan; 4 Faculty of Computer Science, Ruhr-University Bochum, 44801 Bochum, Germany; 5 Department of Cardiology and Angiology, Contilia Heart and Vascular Center, Elisabeth-Krankenhaus Essen, 45138 Essen, Germany

**Keywords:** Coronary angiography, Computed tomography angiography, Radiation exposure, Deep learning

## Abstract

**Objectives:**

Cardiac computed tomography (CT) is essential in diagnosing coronary heart disease. However, a disadvantage is the associated radiation exposure to the patient which depends in part on the scan range. This study aimed to develop a deep neural network to optimize the delimitation of scan ranges in CT localizers to reduce the radiation dose.

**Methods:**

On a retrospective training cohort of 1507 CT localizers randomly selected from calcium scoring and angiography scans and acquired between 2010 and 2017, optimized scan ranges were delimited by two radiologists in consensus. A neural network was trained to reproduce the scan ranges and was tested on two randomly selected and independent validation cohorts: an internal cohort of 233 CT localizers (January 2018–June 2020) and an external cohort from a nearby hospital of 298 CT localizers (July 2020–December 2020). Localizers where a bypass surgery was visible were excluded. The effective radiation dose to the patient was simulated using a Monte Carlo simulation. Scan ranges of radiographers, radiologists, and the network were compared using an equivalence test; likewise, the reduction in effective dose was tested using a superior test.

**Results:**

The network replicated the radiologists’ scan ranges with a Dice score of 96.5 ± 0.02 (*p* < 0.001, indicating equivalence). The generated scan ranges resulted in an effective dose reduction of 10.0% (*p* = 0.002) in the internal cohort and 12.6% (*p* < 0.001) in the external cohort compared to the scan ranges delimited by radiographers in clinical routine.

**Conclusions:**

Automatic delimitation of the scan range can result in a radiation dose reduction to the patient.

**Clinical relevance statement:**

Fully automated delimitation of the scan range using a deep neural network enables a significant reduction in radiation exposure during CT coronary angiography compared to manual examination planning. It can also reduce the workload of the radiographers.

**Key Points:**

*• Scan range delimitation for coronary computed tomography angiography could be performed with high accuracy by a deep neural network.*

*• Automated scan ranges showed a high agreement of 96.5% with the scan ranges of radiologists.*

*• Using a Monte Carlo simulation, automated scan ranges reduced the effective dose to the patient by up to 12.6% (0.9 mSv) compared to the scan ranges of radiographers in clinical routine.*

**Graphical abstract:**

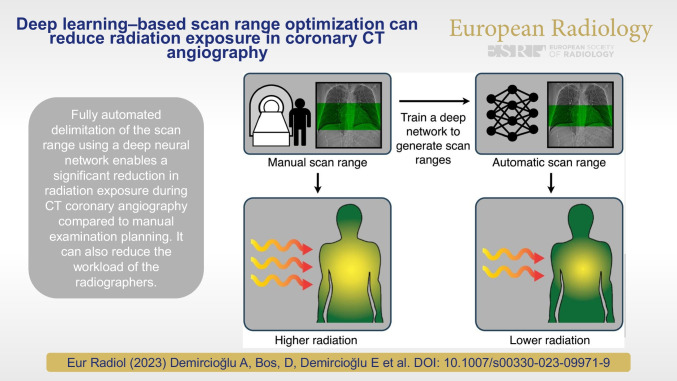

**Supplementary information:**

The online version contains supplementary material available at 10.1007/s00330-023-09971-9.

## Introduction

Chest pain is a common complaint and can be a symptom of coronary artery disease (CAD). Coronary computed tomography angiography (CCTA) can be used to diagnose CAD non-invasively. Although CCTA is affected by several factors, such as image acquisition parameters, increased heart rate, and high coronary calcium burden, CCTA has a very high negative predictive value of 97–99% [1, 2], which can be used to exclude CAD with a very high probability [3, 4]. For this reason, CCTA is recommended as an alternative to invasive angiography in patients with suspected acute coronary syndrome when there is a low-to-moderate pretest probability of CAD and when cardiac troponin or ECG are normal or inconclusive. In patients with stable chest pain, CCTA is recommended by the European Society of Cardiology (ESC) as the first-line diagnostic procedure when there is a low-to-moderate pretest probability of CAD and good image quality of CCTA can be expected [5].

CCTA benefits from being a non-invasive method, giving it an advantage over invasive methods like coronary angiography while at the same time enjoying a low risk of complications. However, during a CCTA, the patient is exposed to radiation, a risk factor for developing cancer [6–8]. Therefore, in clinical routine, the ALARA (As Low As Reasonably Achievable) principle is applied to minimize the potential risks associated with radiation exposure [9]. Different techniques have been developed to reduce the radiation dose of CT, like automated tube current modulation, automated tube voltage selection, iterative image reconstruction, and adaptive dose shielding [10–14].

Consequently, over the recent years the mean effective dose of CCTA declined; for example, Stocker et al report a reduction of 78% between 2007 and 2017 [10] while Schmermund et al report a decline of around 55% between 2009 and 2014 [11].

The scan range is another important factor directly associated with radiation dose in CT [15]. In clinical routine, scan ranges are typically delimited by the radiographers on CT localizers; as such, the scan ranges not only exhibit high intra- and inter-reader variability but since a too narrow scan range would lead to a repetition of the CT scan, radiographers often delimit the scan ranges larger than necessary, resulting in an unnecessary high radiation exposure. Thus, a more optimized and automated scan range is desirable, which reduces the length of the scan range without compromising the image quality and, at the same time, decreases the inter- and intra-reader variability by the radiographers, leading to more homogenous CCTA scans. In addition, such automation would also reduce the workload of the radiographers. Yet, no automation for generating scan ranges for CCTA scans has been developed.

Recently, automation using machine learning, particularly deep learning methods, has become increasingly possible [16]. Therefore, the present study aims to develop and evaluate a fully automated delimitation of the scan range in CCTA using deep learning methods in order to reduce the radiation dose.

## Material and methods

Ethical approval for this retrospective study was granted by the local ethics committee (Institutional Review Board of the University Hospital Essen; registry number 19–8999-BO). Written and informed consent was waived because of the retrospective nature. The study was performed in accordance with all relevant guidelines and regulations.

### Patients

The study population comprised three cohorts: a training, an internal, and an external validation cohort. All cohorts were gathered retrospectively by querying the radiological information systems.

For the training cohort, 2000 CT localizers from either a CCTA or a calcium scoring scan (CACS) were randomly selected from patients who underwent a coronary CT between January 2010 and December 2017. CT localizers of minors (< 18 years) or with a pixel spacing larger than 1.0 mm were excluded to ensure data homogeneity. Only previous bypass surgery was used as an exclusion criterion; patients were not excluded on the basis of other diseases (e.g., scoliosis, kyphosis, etc.). All CT localizers were visually reviewed by two radiologists in consensus (D.B. with 5 years of experience and expertise in radiation safety and K.N. with 20 years of experience and expertise in CCTA) and were excluded if the heart was not fully captured or if the image quality was not considered high enough to delimit the corresponding area, for example if the patient moved during the acquisition.

The internal validation cohort of 500 randomly selected CT localizers was assembled with the same inclusion and exclusion criteria. CT localizers from patients already included in the training cohort were removed. Because the goal was to measure the reduction in radiation dose, localizers from patients without subsequent CCTA were excluded.

Finally, similarly to the internal validation cohort, the external validation cohort was composed of CT localizers from a collaborating hospital (Elisabeth Hospital, Essen, Germany) with the same inclusion and exclusion criteria.

**Fig. 1:**
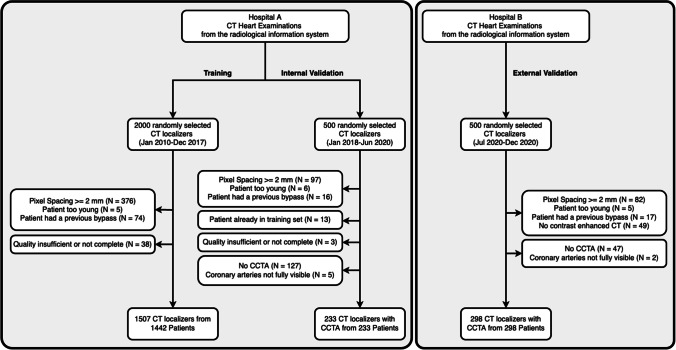
Patient flowcharts with inclusion and exclusion criteria for the model development and internal validation (left) and external validation (right)

In the end, 1507 CT localizers for training, 233 for internal validation, and 298 for external validation were used in the study (Fig. 1).

### Scan acquisition

Acquisition of the CT localizers was performed in inspiration in anterior–posterior direction on several modern, multi-slice CT scanners from Siemens Healthineers (Table 1). Tube voltage was fixed and was between 90 and 120 kV in all cases, while tube currents varied between 20 and 36 mA. For acquiring the CCTA, different examination protocols were used depending on the CT scanner and individual patient characteristics (see Electronic Supplementary Material 1). The acquisition was performed using automated tube current adaption, using primarily a prospective adaptive triggering.

**Table 1:** CT scanners used for the acquisition of the CT localizers and scans. The reference tube voltage and slice collimation refer to the acquisition of the CCTA

	All (*N* = 2038)	Train (*N* = 1507)	Internal validation (*N* = 233)	External validation (*N* = 298)	Reference tube voltage (CCTA)	Slice collimation (CCTA)
SOMATOM Definition Flash	1076	775	3	298	100 kV	2 × 128 × 0.6 mm
SOMATOM Force	503	273	230	0	100 kV	2 × 192 × 0.6 mm
SOMATOM Definition AS/AS +	459	459	0	0	100 kV	128 × 0.6 mm

### Scan range delimitation

**Fig. 2:**
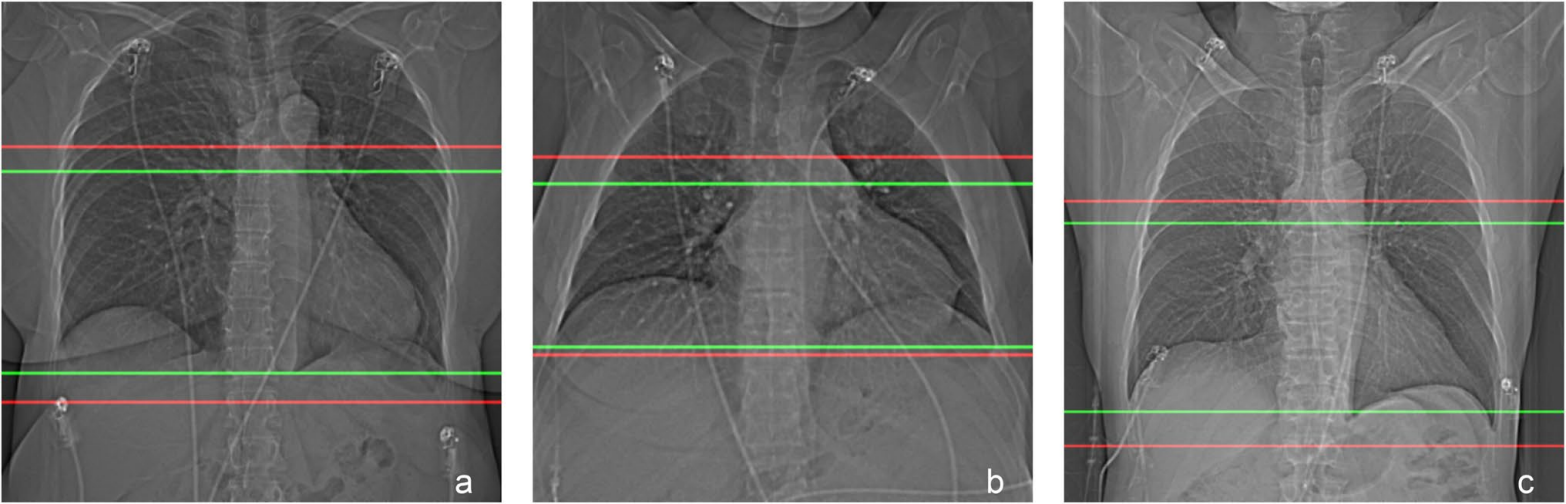
CT localizers for three patients with a scan range from the radiologists. The radiologists’ scan range is depicted in green, and the radiographers in red. **a** The CT localizer of a female patient (55 years). **b** The CT localizer of a male patient (36 years). **c** The CT localizer of a female patient (59 years)

All CT localizers contained the scan ranges delimited by the radiographers in the clinical routine. In addition, the scan ranges previously delimited by the radiographers in the localizers were revised using a custom-tailored tool by expert radiologists in consensus (D.B. and K.N.), whereby the aim was to limit the scan range from the proximal ascending aorta just above the aortic root to the apex of the heart plus a safety margin of 10 mm. Therefore, every localizer had two annotations, one made by the radiographers in clinical routine, one made by the expert radiologists (Fig. 2).

### Assessment of the radiation dose

Radiation doses were reported as effective dose (ED) for the different scan ranges, as defined by the International Commission on Radiological Protection publication 103 [17], and were calculated by a Monte Carlo simulation on a phantom matched to the acquired CT using commercial software (Radimetrics, Bayer Pharmaceuticals). It accounts for patient size by choosing an appropriately sized phantom; it implicitly considers ECG gating by using the CTDI_vol_ readings.

ED was also calculated based on CTDI_vol_ and scan length to compare the more complex Monte Carlo simulation values with a more straightforward estimation. For this, the formula $ED={CTDI}_{vol} \times {scanLength} \times k$ was used, where $k$ is a conversion factor mainly depending on the scanned body part. Following Trattner et al [18], $k$ was set to 0.026 mSv/(mGy × cm). The ED for females were corrected by a factor of 0.045 mSv/(mGy × cm) to account for the breast [19].

### Neural network training

Three different pretrained network architectures were finetuned for the detection of the scan range: Cascade R-CNN [20], VFNet [21], and YOLOX [22]. These networks were chosen since they showed very high performance on several benchmarking datasets. The Cascade R-CNN improves over the often-used Faster R-CNN [23] by employing a multi-stage detector, where each stage refines the predictions of the previous to obtain high accuracy. In contrast, the VFNet is a single-stage detector, but introduces a specific loss-function, to optimize the intersection-over-union-aware classification score, which measures detection and classification accuracy at the same time. In addition, it operates on multiple scales to allow for detection of objects of different sizes. Finally, YOLOX is a single-stage and multi-scale network, but applies many tricks to make the network as fast as possible while still maintaining a high accuracy.

In order to decide which of these networks will perform best, as measured by the highest Dice score, we used a fivefold cross-validation during training. The best-performing model was then evaluated on the internal and external validation cohorts. Details of the training procedure are described in the Electronic Supplementary Material 1, Methods.

### Evaluation

After training the final model, performance was evaluated using the internal and external validation cohorts, neither of which was used for training.

First, the automatically generated scan ranges were quantitatively compared to the radiologists’ scan ranges using five measurements: Dice score, intersection-over-union (IoU) score, and absolute difference (in mm) of the total scan length, and at the upper and lower boundaries. These measures quantify the overlap and difference between the two scan ranges.

Second, the usefulness of the generated scan ranges was qualitatively assessed by determining whether the scan range would completely capture the coronary arteries. For this, the radiologists created in consensus ground truth annotations for each of the CCTA scans in the validation cohorts by annotating the most cranial and caudal slices where the coronary arteries were no longer visible. The evaluation was then performed by computing the accuracy, in other words, by counting how often the coronary arteries were not fully captured in the CCTA scan.

Finally, to test the hypothesis that the generated scan ranges would result in lower radiation exposure to the patients, the estimated radiation doses for the radiographers’, radiologists’, and generated scan ranges were calculated and compared. For this, the effective dose was used as the radiation dose metric. A subgroup analysis was conducted to assess whether the reduction of radiation dose in terms of effective dose was associated with sex.

### Statistical analysis

Descriptive values were reported as reported mean ± standard deviation. χ^2^- and (paired) *t*-tests were used to assess differences between the cohorts. *p* values below 0.05 were considered to indicate a statistically significant difference.

An equivalence test based on a two-sided Wilcoxon signed-rank test with limits of 5 mm was performed to test for the hypothesis that the scan ranges generated by the network are similar to those of the radiologists. The same test with a limit of 2.5 mm was employed to test for equivalence at the upper and lower boundary.

The evaluation of the reduction in radiation exposure is based on the hypothesis that a reduction of at least 5% of the radiation dose exposed to the patient during a CCTA with conventional tube voltage is necessary to be of clinical relevance. Therefore, assuming a mean radiation dose of 10 mSv [15], a superiority test was performed based on a one-sided Wilcoxon signed-rank test with a limit of 0.5 mSv.

Statistical analyses were conducted using the statsmodel library in Python 3.8. All modelling and statistics were performed by a machine learning specialist (A.D. with 15 years of experience in machine learning and computer vision).

## Results

The mean age of all patients was 59.1 ± 13.8 years (range: 18.1–92.3 years), with 819 females and 1154 males (Table 2); a statistically significant difference between the cohorts in age and sex was seen.

**Table 2:** Demographics of the patient collective. Age is reported as mean with standard deviation

	All	Training	Internal validation	External validation
Female	42% (819/1973)	40% (583/1442)	36% (84/233)	49% (146/298)
Male	58% (1154/1973)	60% (859/1442)	64% (149/233)	51% (152/298)
Age [y]	59.1 ± 13.8 (*N* = 2083)	59.8 ± 14.4 (*N* = 1442)	54.2 ± 12.4 (*N* = 233)	59.4 ± 11.2 (*N* = 298)

### Comparison between generated and radiologists’ scan ranges

All networks performed very close during cross-validation (Table 3; Electronic Supplementary Material 1); however, the VFNet performed slightly better than the other two, with a Dice score of 0.966 ± 0.021 vs. 0.965 ± 0.022 for the Cascade R-CNN and 0.963 ± 0.022 for the YOLOX-S network.

**Table 3:** Performance of the networks during the cross-validation, and on the internal and external cohort respectively. The best values during cross-validation are marked with a bold face. Mean differences are measured against the radiologist’s scan ranges. Positive values for mean differences at the upper and lower boundary mean that the networks’ scan ranges were longer, while negative values mean that the networks’ scan ranges were shorter than those of the radiologist

	Dice score	Intersection-over-union	Mean of differences at upper boundary [mm]	Mean of differences at lower boundary [mm]	Mean scan range length [mm]
Cross-validation					
Cascade R-CNN	0.965 ± 0.022	0.932 ± 0.039	1.8 ± 4.5	**0.5 ± 6.4**	125.5 ± 12.3
VFNet	**0.966 ± 0.021**	**0.935 ± 0.038**	1.3 ± 4.5	− 0.7 ± 6.2	123.8 ± 12.4
YOLOX-S	0.963 ± 0.022	0.93 ± 0.041	**0.6 ± 4.8**	− 1.6 ± 6.7	**122.1 ± 11.6**
Internal validation cohort					
VFNet	0.964 ± 0.02	0.93 ± 0.04	− 0.5 ± 4.7	1.1 ± 6.1	119.9 ± 13.1
External validation cohort					
VFNet	0.969 ± 0.019	0.94 ± 0.035	1.1 ± 4.0	− 1.8 ± 4.9	121.0 ± 12.0

**Fig. 3:**
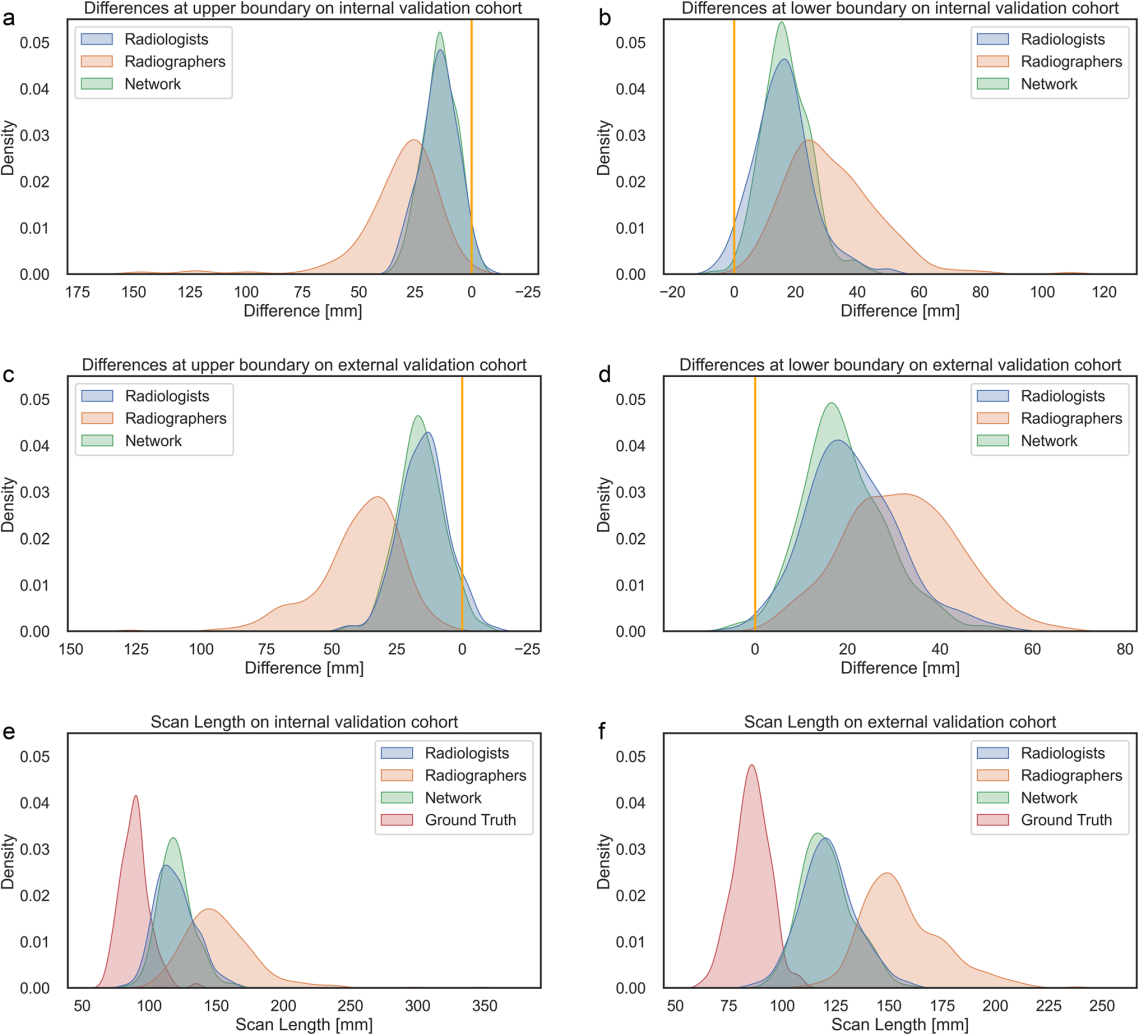
Histogram of the differences at the upper and lower boundary to the ground truth and the overall scan length, measured on both validation cohorts. **a** The differences at the upper boundary evaluated on the internal validation cohort. **b** The differences at the lower boundary evaluated on the internal validation cohort. **c** The differences at the upper boundary evaluated on the external validation cohort. **d** The differences at the lower boundary evaluated on the external validation cohort. **e** The overall scan length in the internal validation cohort for each rater and the ground truth measured from the CT. **f** The overall scan length in the external validation cohort for each rater and the ground truth measured from the CT. In **a**–**d** the ground truth was normalized to have a difference of 0 mm and is denoted by an orange vertical line

Therefore, the VFnet was selected as final model. In the internal validation cohort, it showed a Dice score of 0.964 ± 0.02 and an IoU score of 0.93 ± 0.04 with respect to the radiologists’ scan ranges (Table 3). The mean length of the generated scan ranges was 119.9 ± 13.1 mm (Fig. 3), which was very close to those of the radiologists (119.4 ± 14.6 mm). An equivalence test with a limit of 5.0 mm did not indicate a difference (*p* < 0.001); both scan ranges were significantly shorter than those of the radiographers (134.8 ± 24.9 mm; *p* < 0.001). However, all three were considerably longer than the ground truth scan range, which amounted to 89.2 ± 10.6 mm (*p* < 0.001). At the upper boundary the generated scan ranges were slightly shorter (− 0.5 ± 4.7 mm), while they were slightly longer at the lower boundary (1.1 ± 6.1 mm). An equivalence test with a limit of 2.5 mm showed no statistically significant difference at both boundaries (*p* < 0.001).

On the external cohort, the performance of the network was similar: the Dice score was 0.969 ± 0.019 and an IoU score of 0.94 ± 0.035, while the mean length of the generated scan ranges was 121.0 ± 12.0 mm (Fig. 3), while it was 121.7 ± 12.8 mm for the radiologists (*p* < 0.001), both shorter than those of the radiographers (141.1 ± 18.6 mm). Again, all scan ranges were longer than the ground truth (85.7 ± 8.3 mm; *p* < 0.001). The generated scan ranges were slightly longer at the upper boundary (1.1 ± 4.0 mm) and slightly shorter at the lower boundary (− 1.8 ± 4.9 mm). These differences were not significant under the equivalence test (*p* < 0.001).

### Clinical usefulness

Using the ground truth range in the CCTA scans, the radiologists’ annotations showed an incomplete imaging of cardiac anatomy in less than 6% (Table 4). However, upon inspection, in nearly half of these cases these errors would not have affected the diagnosis (around 3%). The network performed slightly better, and a too short scan range was visible in less than 4% of the CT localizers. After inspection, most of these errors were only marginal and nearly all cases were still clinically useful, resulting in accuracies above 99% (Table 4).

**Table 4:** Number of incomplete CT localizers delineated by the radiologist and the network in both validation cohorts. A CT localizer was considered incomplete if the coronary arteries were not completely imaged in the CCTA. If this had also affected the diagnosis, the CT localizer was considered clinically incomplete

		Upper boundary		Lower boundary	
		Incomplete	Clinically incomplete	Incomplete	Clinically incomplete
Internal validation cohort	Radiologist	8 (96.6%; 225/233)	5 (97.8%; 228/233)	7 (97.0%; 226/233)	0 (100%; 233/233)
	Network	8 (96.6%; 225/233)	2 (99.1%; 231/233)	1 (99.6%; 232/233)	0 (100%; 233/233)
External validation cohort	Radiologist	18 (94.0%; 280/298)	10 (96.6%; 288/298)	2 (99.3%; 296/298)	2 (99.3%; 296/298)
	Network	9 (97.0%; 289/298)	3 (99.0%; 295/298)	4 (98.7%; 294/298)	1 (99.7%; 297/298)

### Dose reduction

**Fig. 4:**
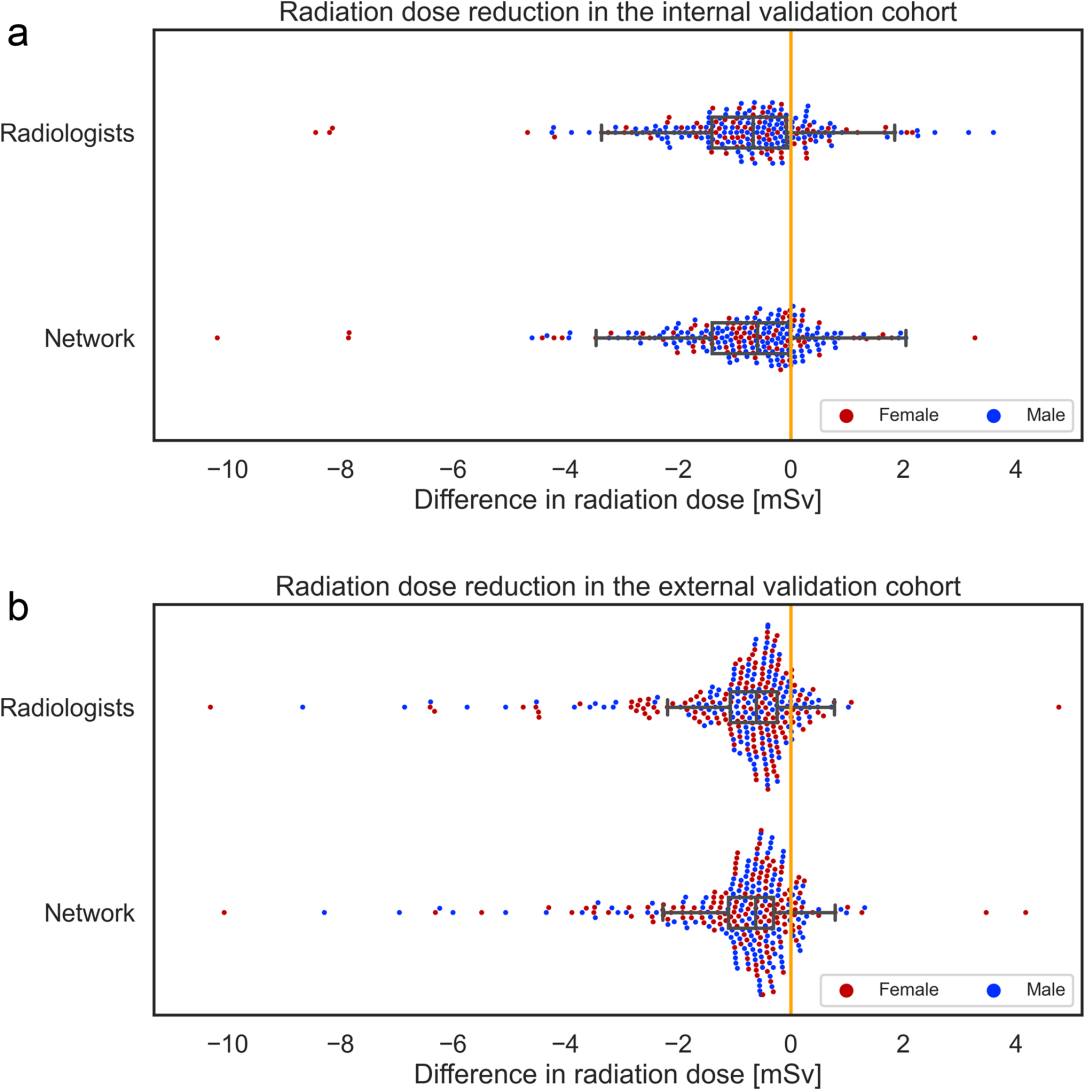
Histograms of radiation dose reductions in terms of effective dose for the radiologists and the network in the internal (**a**) and external (**b**) cohort. Female and male patients are denoted by red and blue dots

The estimated mean effective dose using the Monte Carlo simulation was 8.1 ± 5.0 mSv for the original scan ranges of the radiographers in the internal validation cohort (Fig. 4 and Table 5, see also Electronic Supplementary Material 1). For the radiologists, the simulated effective dose was 7.3 ± 4.4 mSv on average; the network performed virtually the same; dose reductions were 10.1% and 10.0%, respectively. The Wilcoxon signed-rank test for superiority with a limit of 0.5 mSv indicated that reductions were statistically significant (*p* < 0.001 and *p* = 0.002).

**Table 5:** Estimated radiation doses with subgrouping respect to sex. *p* values correspond to a Wilcoxon signed-rank test for superiority. Significant *p* values were marked with a bold face

		All		Female		Male	
		Absolute radiation dose	Dose reduction	Absolute radiation dose	Dose reduction	Absolute radiation dose	Dose reduction
Internal validation cohort	Radiographers	8.1 ± 5.0 mSv	- (reference)	10.5 ± 5.6 mSv	- (reference)	6.7 ± 4.1 mSv	- (reference)
	Radiologist	7.3 ± 4.4 mSv	0.8 mSv (10.1%; ***p* < 0.001**)	9.5 ± 4.8 mSv	1.0 mSv (9.6%; ***p* = 0.009**)	6.0 ± 3.6 mSv	0.7 mSv (10.5%; ***p* = 0.02**)
	Network	7.3 ± 4.4 mSv	0.8 mSv (10.0%; ***p* = 0.002**)	9.5 ± 4.8 mSv	0.9 mSv (8.9%; ***p* = 0.03**)	6.0 ± 3.7 mSv	0.7 mSv (11,0%; ***p* = 0.007**)
External validation cohort	Radiographers	7.5 ± 6.4 mSv	- (reference)	9.6 ± 7.8 mSv	- (reference)	5.2 ± 3.2 mSv	- (reference)
	Radiologist	6.7 ± 5.9 mSv	0.8 mSv (10.6%; ***p* = 0.005**)	8.8 ± 7.2 mSv	0.8 mSv (8.8%; ***p* = 0.01**)	4.5 ± 3.0 mSv	0.7 mSv (14.1%; *p* = 0.07)
	Network	6.5 ± 5.8 mSv	0.9 mSv (12.6%; ***p* < 0.001**)	8.7 ± 7.1 mSv	0.9 mSv (9.6%; ***p* < 0.001**)	4.3 ± 2.4 mSv	1.0 mSv (18.3%; ***p* < 0.001**)

On the external cohort the estimated mean radiation doses using the Monte Carlo simulation were 7.5 ± 6.4 mSv (Fig. 4). The simulated mean radiation dose for the radiologists was 6.7 ± 5.9 mSv, and 6.5 ± 5.8 mSv for the network, corresponding to a dose reduction of 0.8 mSv (10.6%) and 0.9 mSv (12.6%) respectively (Table 5). In both cases the test for superiority was statistically significant (*p* = 0.005 and *p* < 0.001). Similar observations hold for grouping after sex, although higher absolute radiation doses were absorbed by females.

Computing the ED by the formula based on CTDI_vol_ and scan length, the overall radiation dose was higher (11.8 ± 7.2 vs. 8.1 ± 5.0 mSv in the internal and 10.0 ± 7.9 vs. 7.5 ± 6.4 mSv in the external cohort), yet the relative dose reductions were similar (see Electronic Supplementary Material 1).

## Discussion

In this study, we have developed a fully automatic scan range delimitation using a deep neural network to reduce radiation exposure during cardiac CT examinations.

Our study showed that the VFNet is capable of producing scan ranges that are highly accurate and comparable with those expert radiologists, showing a Dice score above 0.95 and a IoU-Score above 0.93. The overall length of the generated scan ranges was very close to those of the radiologists as well, as were the mean differences at both edges. The network did show similarly good results on both the internal and external validation cohorts, demonstrating that it is able to generalize beyond the training set.

Comparing the generated scan ranges with those of the radiographers, a decrease of around 15–20 mm was seen. While the radiographers showed incomplete imaging in 7 cases overall, the radiologist did so in 15 cases. Yet, the network failed only in 7 cases overall, which demonstrates that the network can learn “through the noise” and generate more homogenous and clinically useful scan ranges. In this regard, it performs similarly to the radiographers while still being able to reduce the length of the scan ranges.

Regarding the radiation dose in terms of effective dose, the network was able to reduce it by around 0.8–0.9 mSv (10.6–12.6%) on average when compared to the scan ranges by the radiographers, which is comparable to the estimated dose reduction of the radiologists (0.8 mSv, 10.1–10.6%). The effective dose reductions were shown to be statistically significant when compared to those of the radiographers in clinical routine. Since both show less standard variation when compared to the radiographers, it can be assumed that the generated scan ranges are more homogenous than those of the radiographers; that is, they exhibit much less intra-variability.

We computed the ED using a complex Monte Carlo simulation, but since one can assume that the radiation dose is relatively uniform during the scan, the ED should be related directly to the scan length and could therefore be computed straightforwardly. Indeed, our results showed that the relative savings were comparable, although the direct calculation led to higher overall doses. This difference might be due to the chest-specific conversion factor of 0.026 mSv/(mGy × cm), higher than the previously reported factor of 0.021 [24]. Yet, this higher factor was shown to be more precise [18, 25] and was accordingly adopted in several other studies [10, 26, 27]. Regardless, our method yielded similar relative dose reductions for both methods and thus should be valuable in clinical routine.

We observed an unexpected unequal distribution in sex and age between the training, internal and external validation cohorts. Because the cohorts were consequently enrolled, we suspect that these differences are due to other prospective studies that were conducted at our institute. Regardless, the network showed that it generalizes beyond sex and age, since a similar dose reduction was observed in all cohorts.

As mentioned above, better delimitation of the scan range is not the only way to reduce radiation exposure [28–30]. Hausleiter et al identified several factors influencing the radiation dose in CCTAs [31]. They found that especially tube current modulation and voltage, but also sequential scanning can greatly reduce radiation exposure. These methods can be combined with our automation of scan ranges, leading to a further reduction of radiation dose to the patient. Hausleiter et al also assessed the impact of the scan range on radiation dose, indicating that a 1-cm reduction corresponds to a 5% reduction in radiation dose [15], while more recently, LaBounty et al obtained a 6% reduction [32]. Our results support these analyses and we observed a reduction of 6.6% and 6.3% per 1 cm on the validation cohorts.

Several studies explored the possibility of optimizing the scan range by other means. Jin et al used a CACS instead of the CT localizer to reduce the scan range and showed that this approach could decrease the ED of the CCTA by up to 20% depending on patient characteristics [33]. However, they report the additional radiation exposure from the CACS did not result in lower overall radiation exposure. In contrast, using similar methods, Leschka et al reported an overall dose reduction of around 11% (1.2 mSv) compared to the use of CT localizers [34]. In comparison, Zimmermann et al report a reduction of abound 6% (0.6 mSv) [35]. Young et al showed that the scan length of the CACS can be reduced by including only the proximal and mid-coronary arteries; this way, they reduced the radiation dose of the CACS by 59% and lowered the overall ED [36]. Recently, Duerden et al demonstrated that an ultra-low dose planning scan could be used for scan range delimitation [27]. While the scan length was indeed reduced when compared to the scan length inferred from the CT localizers (117 mm vs. 124 mm; *p* = 0.007), no significant reduction in radiation exposure could be observed. Our approach shows that additional acquisitions like CACS or planning scans could be bypassed entirely if the CT localizer’s scan range is optimized. It could thus yield a reduction in radiation exposure without additional time expenditure.

A major limitation is that the CT localizers were acquired on CT scanners of a single vendor. Thus, it is unclear whether our network generalizes to CT localizers acquired on scanners from other vendors. However, it can be assumed that this will be the case since CT scans measure absolute HU values, which should be very similar across CT scanners. The same should be valid for more modern CT scanners that use a lower tube voltage than our scanners to reduce radiation exposure [10] In this case, we expect that although the overall radiation dose reduction will be lower, the relative reduction will still be comparable.

Another limitation is that the acquisition was performed nearly always with prospective gating. Therefore, the application of our model on retrospective gating needs to be tested. Furthermore, we excluded patients with a coronary artery bypass graft (CABG) because they alter the appearance of the coronary aorta in the localizer. However, since only about 3% of all patients had a CABG, it does not significantly diminish the usefulness of our approach. The network could possibly handle such localizers, but more should be included in the cohorts to allow for successful training.

Although the network was capable of producing scan ranges that reduced the overall radiation exposure to the patient, there is still some room for improvement, since the ground truth scan range was considerably shorter, around 30–35 mm, than the scan ranges of the radiologists and the network. However, due to respiration effects, a certain safety margin must be maintained; otherwise, there is a high risk that the CCTA must be repeated, diminishing the positive effects of the shorter scan ranges.

In conclusion, we presented a fully automated scan range delimitation for cardiac computed tomography angiographies using deep neural networks that leads to a decrease of up to 12.6% of radiation exposure to the patient.

### Supplementary Information

Below is the link to the electronic supplementary material.

Supplementary file1 (PDF 115 kb)

## Abbreviations

CAD: Coronary artery disease
CABG: Coronary artery bypass graft
CACS: Calcium scoring scan
CCTA: Coronary computed tomography angiography
ESC: European Society of Cardiology
ED: Effective radiation dose
IoU: Intersection-over-union

## Declarations

### Guarantor

The scientific guarantor of this publication is Kai Nassenstein.

### Statistics and biometry

Aydin Demircioglu, one of the authors, has significant statistical expertise.

### Study subjects or cohorts overlap

None of the study subjects or cohorts have been previously reported.

### Methodology

• retrospective

• prognostic study

• performed at two institutions
