# Supplementary material for: Deep learning–based scan range optimization can reduce radiation exposure in coronary CT angiography
Source: Eur Radiol. 2023 Aug 8;34(1):411–21. doi: 10.1007/s00330-023-09971-9 (PMC10791769; doi:10.1007/s00330-023-09971-9)
Supplement: Supplementary file 1 — Supplementary file1 (PDF 115 kb) [file 330_2023_9971_MOESM1_ESM.pdf]

<u>ELECTRONIC SUPPLEMENTARY MATERIAL</u>

Deep learning-based scan range optimization can reduce radiation exposure

Methods

Scan acquisition and parameters

For acquiring the CTTA, different examination protocols were used depending on the CT scanner and individual patient characteristics. Patients examined underwent primarily a prospective adaptively triggered sequential (quality reference mAs: 370 mAs (Flash)/300 mAs (Force)). In a few cases (N = 13 in the validation set, none in the test set) a retrospective gated CCTA was used.

The tube voltage was fixed between 90-120 kV. The collimation size was 128x0.6mm, and the rotation speed was 0.28/s. All examinations on dual-source CT scanners were performed in single-source mode, and automated tube current modulation (Siemens CARE Dose 4D, Siemens Healthineers) was used.

Preprocessing

All CT localizers were retrieved anonymously and then converted to 8-bit PNGs for better handling. All images had a fixed resolution of 512x512 pixels. They were then converted to RGB using the OpenCV library by applying a contrast-limited adaptive histogram equalization (CLAHE) with three different clipLimits and tileSizes (red channel: clipLimit = 64, tileSize=1; green channel: clipLimit = 32, tileSize=2; blue channel: clipLimit = 16, tileSize=4). The images

were then cut to a width of 384 since there was no important information at the left and right border.

Neural Network architectures

This study employed three well-known and commonly used localization networks: the Cascade R-CNN, the VFNet, and the YOLOX-S. These three were chosen because they have been shown to produce excellent results in previous studies. The implementations from the MMDetection 2.20 library [1] were used for development. All networks were pre-trained using the COCO dataset [2] and fine-tuned on the annotated scan ranges. Fine-tuning was performed by using the same learning rate and optimizer that were used during pre-training; the corresponding settings can be found in the MMDetection framework (see the configurations "cascade_rcnn_x101_64x4d_fpn_1x_coco.py" for Cascade R-CNN, "vfnet_x101_64x4d_fpn_mdconv_c3-c5_mstrain_2x_coco.py" for the VFNet and "yolox_s_8x8_300e_coco.py" for YOLOX-S). Several augmentations were employed:

- Random cropping to a height of 480
- Random changes to the brightness, contrast, and saturation (+/- 20%)
- Adding gaussian noise
- Applying an elastic transformation
- Applying a random horizontal flip

Images were then normalized to the intensity mean and standard deviation of the COCO dataset. The network was developed using Python 3.8.

The best network was determined by employing a 5-fold cross-validation and using the Dice scores between the generated and the radiologist's scan ranges as measure.

Evaluation

After the best network structure was determined, the network was retrained using all training data because, in general, neural networks benefit from more data. The retrained model was considered to be final. Its performance was then evaluated on the two independent validation data sets. This final evaluation took place only once to avoid introducing a bias by repeatedly optimizing for the validation set, which would lead to severe overfitting.

Direct computation of the effective radiation dose

The effective radiation dose was also computed using the formula $ED = CTDI_{vol} \times DLP \times k$. Here k is a conversion factor that needs to be chosen; we used k=0.026, since this value was proposed in a recent study by Trattner et al. [3]. However, for females this factor needs to be corrected. From Deak et al. [4] we deduced that the correction factor should be increased by 75% (see Figure 4, chest in [4]). Thus, for females we used k=0.045. Results are in Table S1.

References

1. Chen K, Wang J, Pang J, et al (2019) MMDetection: Open MMLab Detection Toolbox and Benchmark. ArXiv190607155 Cs Eess

2. Lin T-Y, Maire M, Belongie S, et al (2015) Microsoft COCO: Common Objects in Context. ArXiv14050312 Cs

3. Trattner S, Halliburton S, Thompson CM, et al (2018) Cardiac-Specific Conversion Factors to Estimate Radiation Effective Dose From Dose-Length Product in Computed Tomography. JACC Cardiovasc Imaging 11:64–74. https://doi.org/10.1016/j.jcmg.2017.06.006

4. Deak PD, Smal Y, Kalender WA (2010) Multisection CT Protocols: Sex- and Age-specific Conversion Factors Used to Determine Effective Dose from Dose-Length Product. Radiology 257:158–166. https://doi.org/10.1148/radiol.10100047

		All		Female		Male	
		Absolute Radiation Dose	Dose Reduction	Absolute Radiation Dose	Dose Reduction	Absolute Radiation Dose	Dose Reduction
Internal validation cohort	Radiographers	11.8 ± 7.2 mSv	- (Reference)	15.1 ± 8.2 mSv	- (Reference)	9.9 ± 5.8 mSv	- (Reference)
	Radiologist	10.5 ± 6.1 mSv	1.3 mSv (10.9%; **p<0.001**)	13.1 ± 6.6 mSv	1.9 mSv (12.7%; **p<0.001**)	9.0 ± 5.3 mSv	0.9 mSv (9.4%; **p<0.001**)
	Network	10.5 ± 6.1 mSv	1.3 mSv (10.8%; **p<0.001**)	13.2 ± 6.5 mSv	1.8 mSv (12.1%; **p<0.001**)	8.9 ± 5.2 mSv	1.0 mSv (9,8%; **p=0.001**)
External validation cohort	Radiographers	10.0 ± 7.9 mSv	- (Reference)	12.8 ± 9.7 mSv	- (Reference)	7.1 ± 3.9 mSv	- (Reference)
	Radiologist	8.9 ± 7.2 mSv	1.2 mSv (11.7%; **p<0.001**)	11.4 ± 8.8 mSv	1.4 mSv (11.2%; **p<0.001**)	6.2 ± 3.6 mSv	0.9 mSv (12.5%; **p<0.001**)
	Network	8.7 ± 7.0 mSv	1.4 mSv (13.5%; **p<0.001)**	11.2 ± 8.6 mSv	1.6 mSv (12.2%; **p<0.001**)	6.0 ± 3.1 mSv	1.1 mSv (16.0%; **p<0.001**)

Table S1: Estimated radiation doses with subgrouping respect to sex. P values correspond to a Wilcoxon signed-rank test for superiority. Significancy is marked with a bold face.
